# The Book World of Medicine and Science

**Published:** 1899-09-02

**Authors:** 


### THE BOOK WORLD OF MEDICINE AND SCIENCE.

SELBY: A PATHOLOGICAL MORALITY. By EPPIE FRAZER. (London: J. Bale, Son, and Danielsson. 1899. Pp. 99. Price 7s. 6d. net.)

"SELBY" is not easily characterised. That it is clever, intensely clever, we make no doubt. But the "pathological morality" reminds us just a little of the "Chinese Metaphysics" of the *Eatanswill Gazette* Like the young lion of that famous periodical, the author of "Selby" has "combined his information." The fact seems to be that the author, who happens to be acquainted with matters medical, has wished to treat of certain problems, elemental and unsolved, perhaps insoluble. To this end he has chosen for his puppets two medical men and two nurses. His background and mechanism are pathological, his medium, verse. Of the puppets, one—a matron—has been not unfairly called a Christian sensualist; another—Mrs. Bubb, a nurse—is also a sensualist, but lacking a counterpoise of piety. One medical man wears knickerbockers, and seeks to subjugate his fleshy self. We seem to know him. Perhaps in the poem, as in real life, it is a lack of virility that compels his ideal. The other medical man, having sown his wild oats and garnered a full crop, devotes himself to "the quest of the crab"—the solution of the cancer problem. In the end he alone survives. The three others, with pathological truth if not artistic conventionality, being disappointed, the two nurses in their desires and the man in his ideal, virtually commit suicide. The pathologist alone, as we have said, remains; a nineteenth century Prometheus. But the merit of "Selby" is in the versification. This is exceedingly striking. And, in particular, the author has succeeded in adapting his metres to the speakers and the speech.

The descriptive portions are less happy. Old Dalgin, "doting o'er her past renown," is but a feeble shadow of Madras, as Kipling has it, "brooding o'er ancient fame." The matron's lyrical outburst, "Love is a clambering plant," is, however, in spite of its frankness, really a gem. And in the lines beginning, "Happy the woman of old," there is more than a touch of sacred fire. Many of the couplets and single lines are exceedingly effective, and the more musical of them haunt the ear. In fine, we congratulate the author, but on his poetry rather than his "pathological morality."

A MANUAL OF PHYSIOLOGY. By LEONARD HILL, M.B. (London: Ed. Arnold. 1899. Pp. 483. Illustrated. Price 6s.)

MR. LEONARD HILL has, as he says, set himself in this little book to give the general reader some insight into the structure and functions of living man. Within his limits he has certainly succeeded. The ground covered is practically that of Huxley's classic primer; and Mr. Hill has achieved a simplicity of style which mingles pleasantly with the wealth of allusion and literary flavour that may justly be expected from the son of Dr. Birkbeck Hill. At the same time, we are bound to note an occasional ambiguity of expression and a regrettable laxity in methods of punctuation, while on page 6, line 7, there is to be found a remarkable misprint. At times Mr. Hill becomes perhaps unduly didactic, and wanders, not altogether fortunately, into alien paths. For instance, he condemns the Italian Government for the matter of salt-taxation. Are our withers unwrung, or does not Mr. Hill know how the inland revenue of British India is maintained? Mr. Hill says that there are very few persons who can maintain their health on a strictly vegetable diet. Surely it is on a purely vegetable diet that many millions of Orientals support learned and laborious lives. Physiological dicta are very well, and Mr. Hill confidently relegates beef-tea and meat juice to the limbo of "flavouring agents." But we fancy that a good many practitioners do, and will continue to employ these solutions of peptones and cardiac stimulants with advantage on appropriate occasions. Mr. Hill's book is, on the whole, so excellent that we regret these and other little excrescences and excursions. In the latter chapters Mr. Hill falls into a curious but not infrequent error. "Tone" is a certain quality of certain sounds; "a tone" is the "interval" between certain sounds. But no musician would, we hope, constantly confuse "a" tone with "a" note, as Mr. Hill does. No doubt in every-day speech such phrases as "the tones of a man's voice" are used when "sounds" are referred to. But in a scientific work by "a" tone we expect a musical interval, and not a sound or note to be meant.

ESSENTIALS OF MODERN TREATMENT OF DISEASE. By K. M. NADKARNI. (Vepery: N. K. Rao. London: Baillière, Tindall, and Cox. 1898. Vols. II. Pp. 462.)

THIS little work, written by a native physician, printed and published by native firms, is praiseworthy and peculiarly interesting. It is of course a compilation, but it is a compilation made with intelligence and usefully arranged. The form is that of a dictionary, and the appropriate treatment for each disease is found on referring to the name of the disease. The information given seems to us accurate. It has evidently been drawn from the latest channels of cosmopolitan therapeutics. Some of the authorities quoted are not, it is true, well-known in England, and some of the methods advised are not for universal acceptance. For greater convenience the volumes are interleaved with blank pages; heavy type is judiciously made use of where emphasis is required. Mr. Nadkarni may on the whole justly contemplate his little work with satisfaction.

ANNUAL REPORT OF PROCEEDINGS UNDER THE DISEASES OF ANIMALS ACT, 1898. (London: Darling and Son, 1899. Pp. 104. Price 6½d.)

IN the information furnished us by this report we have some evidence of the great and benevolent activity of the latest Government department—the Board of Agriculture. It is gratifying to learn that, as a result of the last few years' activity, Great Britain is practically free from any contagious disease of cattle—that at the present time cattle plague, foot and mouth disease, and pleuro-pneumonia are non-existent. It is also good hearing that agriculturists, instead of resisting, eagerly invoke State interference in these matters. In fact, the too great readiness of farmers and stockholders to certify certain ailments as swine fever is actually a matter of official complaint. Glanders is another disease almost stamped out. At present it is practically confined to Middlesex and Lanark. Mallein, as a diagnostic agent, continues to deserve the veterinarian's attention. The statistical tables are very interesting. We learn from them that the actual number of living cattle, swine, and sheep in Great Britain continues to increase. More cattle are, we see, imported than formerly from Ireland, but fewer swine. Many other points of interest in this valuable report might be alluded to. We must content ourselves, however, with saying that it is a striking monument of the beneficent effect of certain forms of State interference.

